# Computational Optimization of Sandwich Silicone Rubber Composite for Improved Thermal Conductivity and Electrical Insulation

**DOI:** 10.3390/polym16050616

**Published:** 2024-02-23

**Authors:** Abdulrahman A. Alghamdi

**Affiliations:** Mechanical Engineering Department, College of Engineering and Architecture, Umm Al-Qura University, Makkah 24382, Saudi Arabia; aasghamdi@uqu.edu.sa; Tel.: +966-555531140

**Keywords:** silicone rubber composite, thermal conductivity, electrical resistivity, multiscale finite element

## Abstract

The efficient dissipation of heat has emerged as a crucial concern for modern electronic devices, given the continuous increase in their power density and consumption. Thus, the utilization of thermally conductive but electrically insulating silicone rubber composites as a thermal interface material has garnered significant interest. In this study, the effects of the filler volume fraction, filler orientation, layer volume fractions, layer configuration, and a number of layers on the thermal conductivity and electrical resistivity of silicone rubber composites were examined using a multiscale finite element modeling strategy. The results demonstrated that modification of the filler orientation can change the thermal conductivity by 28 and 21 times in the in-plane and through-thickness directions, respectively. The in-plane thermal conductivities of silicone rubber/boron nitride and silicone rubber/expanded graphite layers exhibit a percolation phenomenon at filler volume fractions of 35% and 30%, respectively. The electrical resistivity of the composite increases exponentially with a decrease in the number of layers.

## 1. Introduction

The efficient dissipation of heat generated by electronic devices is a subject of considerable interest in power, communication, and information technologies [1]. Heat dissipation is crucial for ensuring optimal electronic device performance, durability, and reliability, especially as devices become increasingly integrated and miniaturized [2,3,4]. A promising approach for addressing thermal challenges is the development of thermal interface materials (TIMs) with exceptional thermal conductivity and electrical insulation capabilities. Another key aspect in the development of TIMs is the ability to securely join layers with significantly different coefficients of thermal expansion (CTEs). Soft materials are extensively used for thermal management in electronic devices owing to their ease of processing, cost-effectiveness, and weight [5,6,7]. Among these materials, silicone rubber (SR) is considered a promising candidate for flexible TIMs owing to its high elasticity and electrical resistivity [8,9,10,11]. Additionally, the flexibility of SR allows it to minimize internal stress and ensure the integrity of electronic components across a broad temperature range [12]. However, the intrinsic low thermal conductivity of SR and its composites significantly limits the use of these materials as TIMs in modern electronics [13].

Many researchers have attempted to enhance the thermal conductivity of SR composites by introducing different thermally conductive fillers using various methods. Initial studies focused on ceramic filler materials that are thermally conductive and electrically insulating, such as boron nitride (BN) [11,14,15], alumina nitride (AlN) [16,17], silicon carbide (SiC) [8,18,19], and aluminum oxide (Al_2_O_3_) [20,21,22]. However, traditional processing methods often result in a random filler distribution within the SR matrix, leading to limited improvement in the thermal conductivity even with high filler volume fractions, which compromises the processability and flexibility of SR composites [23,24]. To address this problem, many processing methods have been developed to control the filler orientation to enhance the thermal conductivity of polymer composites, especially SR composites. These methods involve the use of shear force [25,26], electric [27], or magnetic fields [28,29] to align fillers in a specific orientation, creating effective pathways for heat dissipation through conduction with minimum filler volume fraction. Typically, the shear force technique is widely used owing to its compatibility with most filler materials, along with its ability to provide a high degree of alignment and uniform distribution of the filler in a polymeric matrix [26]. Combining these techniques with ceramic filler materials enables the production of polymer composites with high electrical insulation properties and moderate thermal conductivities [30]. Recently, carbonaceous fillers with ultrahigh thermal conductivity, such as graphene, carbon nanotubes (CNTs), expanded graphite (EG), and graphite particles, have been reported to significantly enhance the thermal conductivity of SR composites [31,32]. However, these carbon-based fillers possess a high electrical conductivity, which may result in the degradation of the electrical insulation capabilities of the composites.

Various strategies have been established to address this problem, with the layer-by-layer (LBL) assembly technique being a representative technique that can produce laminated nanocomposites with excellent thermal conductivity and electrical insulation. The composites fabricated using LBL exhibit planar alignment and well-organized multilayer structures, facilitating efficient heat dissipation at low filler volume fractions [33,34]. For example, Zhang et al. [35] prepared high-density polyethylene (HDPE) multilayer composites reinforced with BN and carbon-based fillers. These composites, fabricated through melt extrusion, exhibited a laminated structure (HDPE/BN/multiwall CNTs/graphite) involving 2–32 layers, with a through-thickness thermal conductivity of 1.45 W/m-K. Additionally, Feng et al. [23] fabricated a laminated structured composite SR/graphene nanoparticles/BN using a two-roll mill extrusion method, resulting in a significant increase in thermal conductivity to 8.45 W/m-K. Song et al. [34] developed hybrid films of cellulose/graphene nanosheets with highly anisotropic thermal conductivity, reaching an in-plane thermal conductivity of 12.6 W/m-K after 40 assembly cycles. Xue and colleagues [11,31,36] conducted multiple studies on enhancing the thermal conductivity of SR-based composite using the LBL technique. The resulting composites exhibited outstanding electrical insulation properties and highly anisotropic thermal conductivity, with in-plane and through-thickness thermal conductivity values of 23.4 and 0.7 W/m-K, respectively.

The reliability and precision of the finite element (FE) approach have been demonstrated in numerous studies. This approach can reduce experimental effort, thereby alleviating related costs and time. Therefore, FE analyses have been extensively applied to optimize the polymer matrix composite design. For example, Sun et al. [37], Ding et al. [38], and Khosravani et al. [39] focused on predicting the thermal properties of conductive polymeric composites. Matos et al. [40] and Wang et al. [41] used FE models to optimize electrical properties. These strategies could facilitate the prediction of homogenized properties and provide guidance for the optimal design of novel composites. However, only a few researchers have focused on using FE techniques to examine the thermal and electrical properties of polymeric composites. For example, Li et al. [42] performed such analyses on thermally and electrically conductive polymer composites.

Considering these aspects, this study aimed to develop reliable and accurate multiscale FE models to predict the thermal conductivity and electrical resistivity of multilayer SR/BN-SR/EG composites that are thermally conductive and electrically insulating. The multiscale FE approach was used to investigate the effects of various parameters of filler materials, i.e., the type, volume fractions, orientation, and arrangement, on the thermal conductivity and electrical insulation of the SR composite. The FE results were validated against previously reported experimental results.

## 2. Materials

**Figure 1:**
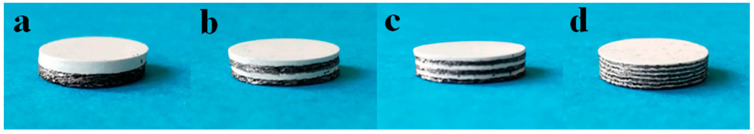
Images of SR/BN-SR/EG *samples* with (**a**) 2, (**b**) 4, (**c**) 6, and (**d**) 12 layers. Adapted with permission from [36], Elsevier, 2024.

The investigated composite, developed by Xue et al. [36], consists of multiple layers of alternately stacked SR/BN and SR/EG. The volume fraction of the BN platelets in SR/BN was 0.395, with an average diameter and thickness of 10 µm and 300 nm, respectively. The volume fraction of the EG platelets in SR/EG was 0.35, with an average diameter and thickness of 20 µm and 100 nm, respectively. Each layer of SR/BN and SR/EG was prepared separately through multiple runs of a two-roll mill. The fillers BN and EG were mixed with SR and aligned in one direction. The resulting sheets were precisely cut into various pieces. The SR/BN and SR/EG pieces were stacked alternately and placed in a hydraulic hot press to cure for 20 min at 170 °C and 15 MPa. Figure 1 shows the SR/BN-SR/EG composite with different numbers of layers.

The thermal conductivity (*K*) was measured through an established protocol [36] using a thermal constant analyzer (TPS3500, Hot-Disk, Göteborg, Sweden) under normal room-temperature conditions. A probe, serving as both a heater and temperature sensor, was placed between two planar samples of a specific size. The volume resistivity was determined using a direct-reading electrometer along with a test fixture at room temperature, with a DC voltage of 400 V. The electrical conductivity was measured using a precision impedance analyzer.

## 3. FE Modeling

### 3.1. Multiscale FE Modeling

A two-scale FE modeling approach was introduced to predict the thermal and electrical conductivities of the SR/BN-SR/EG composite. First, the thermal and electrical conductivity values of the SR/BN and SR/EG layers were separately predicted using a hexagonal filler unit-cell model. Second, the predicted thermal and electrical conductivities of the SR/BN and SR/EG layers were applied to the composite unit cell, and the obtained results were compared with the experimental data reported by Xue et al. [36]. The ABAQUS software [43] package was used to perform FE modeling. The steps are described in the following text.

**Figure 2:**
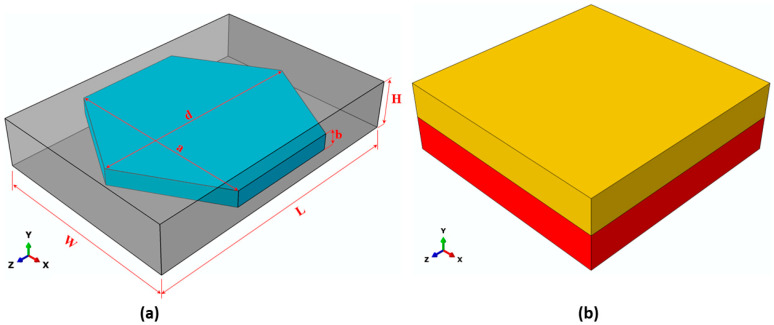
(**a**) Hexagonal filler unit cell and (**b**) composite unit cell.

**Figure 3:**
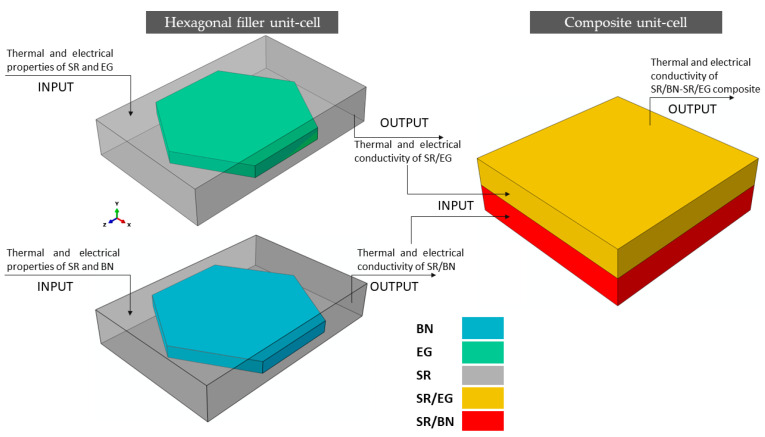
Multiscale FE modeling of thermal and electrical conductivity of the SR/BN-SR/EG composite.

To predict the thermal and electrical conductivity of the composite, it was necessary to individually predict the effective thermal and electrical conductivity values of the SR/BN and SR/EG layers. To simplify the FE modeling of these layers, the BN and EG fillers were assumed to have regular hexagonal platelet shapes. Additionally, the fillers were assumed to be uniformly distributed with a periodic pattern in the SR matrix. Consequently, a unit cell was constructed for these fillers, featuring a regular hexagonal platelet filler surrounded by a rectangular SR matrix (Figure 2a). Similar unit cells have been used in previous studies [44,45] to model polymer composites reinforced with BN. Owing to the differences in the diameter, thickness, and volume fraction of BN and EG, the dimensions of the hexagonal filler unit cell of the SR/BN layer were different than those for the SR/EG layer, as outlined in Table 1. The thermal and electrical conductivities predicted from the hexagonal filler unit cell of the SR/BN and SR/EG layers were then applied to the composite unit cell consisting of two homogenous layers of SR/BN and SR/EG (Figure 2b). The thermal and electrical conductivity values obtained from the hexagonal filler unit-cell models of SR/BN and SR/EG were assigned to the first and second layers, respectively. Figure 3 illustrates the process flow of the multiscale FE modeling technique adopted in this study.

### 3.2. Material Properties of the Constituents

Table 2 summarizes the input thermal and electrical properties of SR, BN, and EG.

### 3.3. Boundary Conditions

The FE computations in this work involved two types of analyses: steady-state thermal analysis, yielding thermal conductivity, and steady-state electrical analysis, yielding electrical conductivity and resistivity. The thermal and electrical properties listed in Table 2 were assigned to the hexagonal filler unit-cell model. Both thermal and electrical steady-state analyses were performed on the hexagonal filler unit-cell model to determine the in-plane and through-thickness thermal and electrical conductivities of the SR/BN and SR/EG layers. The thermal and electrical conductivity values obtained from the hexagonal filler unit-cell model were then incorporated into the composite unit cell to calculate the thermal and electrical conductivities of the composite SR/BN-SR/EG through similar thermal and electrical steady-state analyses.

#### 3.3.1. Thermal Boundary Conditions

**Figure 4:**
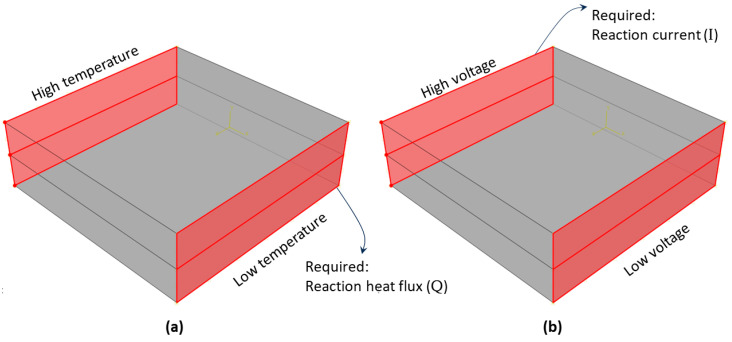
Boundary conditions: (**a**) thermal and (**b**) electrical.

The thermal FE analysis required the application of a temperature difference Δ*T* (temperature gradient) between two opposing faces perpendicular to any spatial direction while the other faces remained thermally isolated. Figure 4a illustrates the thermal boundary conditions in the composite unit-cell model. These boundary conditions were used to predict the heat conductivity in the in-plane direction. The back surface was exposed to a high temperature of 100 °C, whereas the front surface was exposed to a lower temperature of 0 °C. The temperature differential between these two opposing surfaces induced heat flux *Q* directed toward the surface with a lower temperature. The total heat flux was computed using ABAQUS by summing the reaction heat flux values for all nodes on the front surface with a lower temperature. After determining *Q*, the thermal conductivity *k* was estimated using Fourier’s law [50]:

(1) $$k=-\frac{Q\times L}{A\times ∆T}$$

where *L* is the distance between the two parallel surfaces where the temperature gradient is applied, and *A* is the cross-sectional area of the surface with a lower or higher temperature.

The through-thickness thermal conductivity was simulated using the same boundary conditions, albeit on the top and bottom surfaces. The boundary conditions were applied twice, in the in-plane and through-thickness directions, to each scale model (hexagonal filler unit cell and composite unit cell).

#### 3.3.2. Electrical Boundary Conditions

The electrical FE analysis involved the application of a voltage difference Δ*V* (potential difference) between two opposing faces perpendicular to any spatial direction, with the remaining faces being electrically isolated. Figure 4b illustrates the electrical boundary conditions in the composite unit cell model. These boundary conditions were used to predict the electrical resistivity in the in-plane direction. The back surface was exposed to a high voltage of 10 V, whereas the front surface was exposed to a lower voltage of 0 V. The voltage differential between these two opposing surfaces induced an electric current (*I*) directed toward the surface with a lower voltage. The electric current was computed using ABAQUS by summing the reaction electric current values for all nodes on the back surface, corresponding to the high-voltage surface. After determining *I*, the electrical resistance *R* was estimated using Ohm’s law:

(2) $$R=\frac{∆V}{I}$$

Finally, the electrical conductivity σ was determined using Pouillet’s law [51]:

(3) $$\sigma =\frac{1}{R}\times \frac{l}{A},$$

where *L* is the distance between the two parallel surfaces where voltage is applied, and *A* is the cross-sectional area of the surface with a lower or higher voltage. The electrical resistivity ρ was defined as the reciprocal of the electrical conductivity [51]:

(4) $$\rho =\frac{1}{\sigma }$$

The through-thickness electrical resistivity was simulated using the same boundary conditions, albeit on the top and bottom surfaces. The boundary conditions were applied twice, in the in-plane and through-thickness directions, to each scale model (hexagonal filler unit cell and composite unit cell).

## 4. Results and Discussion

### 4.1. Validation of FE Modeling Approach

The multiscale FE modeling approach adopted in this study first used the hexagonal filler unit cell to determine the thermal conductivities of the SR/BN and SR/EG layers. Table 3 lists the thermal conductivity values in the three orthogonal directions. Next, the through-thickness and average in-plane thermal conductivity values of the SR/BN and SR/EG layers were assigned to the composite unit-cell model. The through-thickness and in-plane thermal conductivities of the composite unit cell model are listed in Table 4, along with the experimental results obtained by Xue et al. [36]. 

The predicted through-thickness and in-plane thermal conductivity values of the SR/BN-SR/EG composites were 25.71% and 7.31% higher than the experimental values, respectively. These differences were attributable to the oversight of porosity, particularly cracks at the filler/matrix interface, which significantly influence the thermal and electrical conductivities in the through-thickness direction. Another potential reason for these discrepancies could be the assumption of a uniform and periodic distribution of the fillers. Despite these differences, the results obtained using the multiscale FE modeling approach were consistent with the experimental data.

Similar to the thermal analysis, the multiscale FE modeling approach first used the hexagonal filler unit cell to determine the electrical resistivity of the SR/BN and SR/EG layers. Table 5 lists the electrical resistivity values in the case of continuous and discontinuous fillers. Next, the electrical resistivity values for the SR/BN and SR/EG layers were assigned to the composite unit-cell model. The electrical resistivity of the composite unit cell model was predicted twice, once with continuous fillers and once with discontinuous fillers. The results are presented in Table 6. Xue et al. [36] experimentally measured the electrical resistivity of the SR/BN-SR/EG composite consisting of two layers to be 1.92 × 10^14^ Ω·cm, consistent with the FE result of the composite unit cell with a continuous filler (1.4 × 10^14^ Ω·cm). Therefore, for the subsequent analysis of the electrical resistivity, continuous filler material was considered in this work.

### 4.2. Effects of Filler Volume Fractions

Two approaches were used to investigate the effect of filler volume fractions on the thermal conductivity and electrical resistivity of the composite. In the first approach, the volume fractions of the fillers within the SR/BN and SR/EG layers were maintained constant while the proportion of each layer was altered. In the second approach, the proportions of the SR/BN and SR/EG layers were maintained, and the volume fractions of the fillers within these layers were modified.

**Figure 5:**
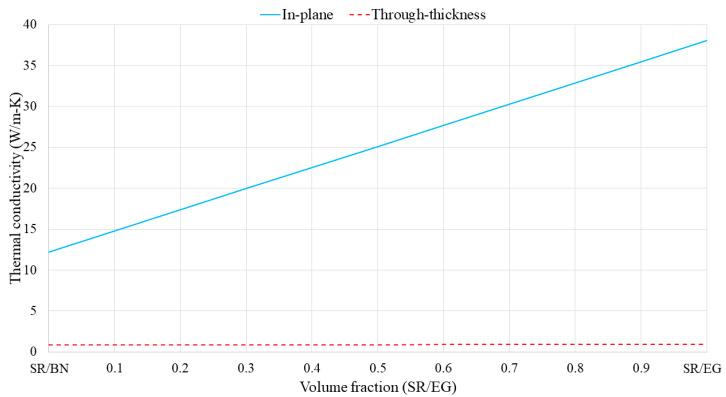
Thermal conductivity of SR/BN-SR/EG composite as a function of the SR/EG volume fraction.

**Figure 6:**
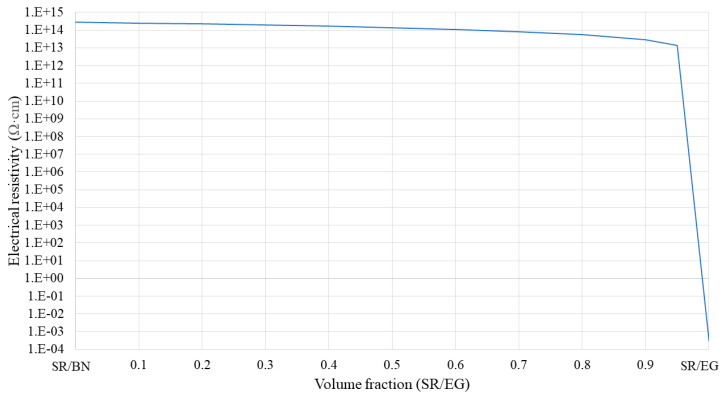
Electrical resistivity of SR/BN-SR/EG composite as a function of the SR/EG volume fraction.

Specifically, in the first approach, the volume fractions of BN and EG in the SR/BN and SR/EG layers were set as 0.395 and 0.35, respectively. Figure 5 shows the through-thickness and in-plane thermal conductivities of the SR/BN-SR/EG composite as a function of the SR/EG volume fraction. The through-thickness and in-plane thermal conductivities exhibited a linear relationship with the SR/EG volume fraction. The change in the through-thickness direction was minimal compared with that in the in-plane direction owing to the high anisotropy of both layers. Figure 6 shows the electrical resistivity of the SR/BN-SR/EG composite as a function of the SR/EG volume fraction. The SR/BN layer functioned as an electrical insulator, and the reduction in the electrical resistivity of the composite caused by the decrease in the SR/BN volume fraction did not exceed two orders of magnitude. Even with the minimum volume fraction of the SR/BN layer (0.05), the electrical resistivity of the composite was still higher than the critical resistance for electrical insulation (10^9^ Ω·cm) [52].

**Figure 7:**
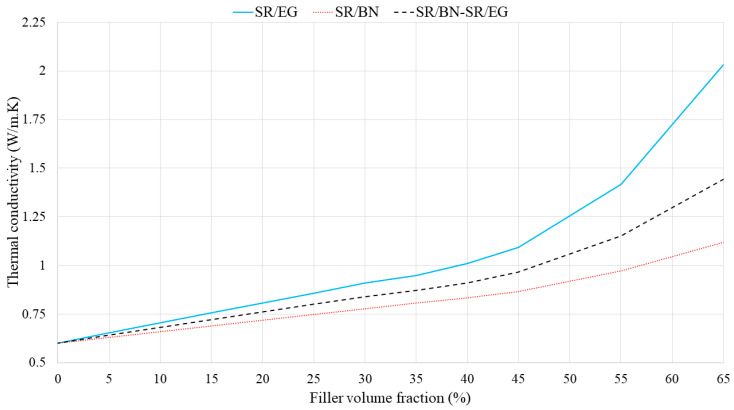
Through-thickness thermal conductivities of SR/BN, SR/EG, and SR/BN-SR/EG composites as a function of the filler volume fraction.

**Figure 8:**
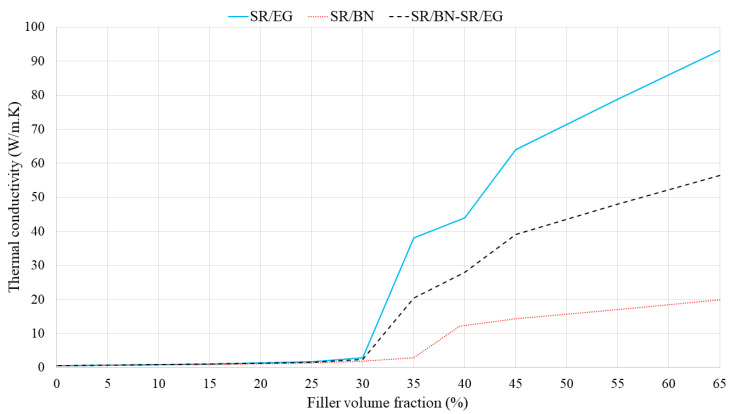
In-plane thermal conductivities of SR/BN, SR/EG, and SR/BN-SR/EG composites as a function of the filler volume fraction.

In the second approach, the volume fraction of both the SR/BN and SR/EG layers was set as 0.5. Figure 7 shows the through-thickness thermal conductivities of SR/BN, SR/EG, and SR/BN-SR/EG composites as a function of the filler volume fraction in each layer. In all three cases, the through-thickness thermal conductivities increased linearly until the volume fraction reached 45%, after which an exponential increase was observed. At the maximum filler volume fraction (65%), the through-thickness thermal conductivities of the SR/BN, SR/EG, and SR/BN-SR/EG composite samples improved by 233%, 86%, and 141%, respectively. Notably, EG had a more significant effect than BN owing to the higher through-thickness thermal conductivity. Figure 8 shows the in-plane thermal conductivities as a function of the volume fraction of each layer. The in-plane thermal conductivities of SR/BN and SR/EG exhibited a percolation phenomenon, showing a sharp increase at a critical filler volume fraction owing to the formation of a filler network [53]. Several studies have demonstrated the occurrence of the percolation phenomenon in composite materials reinforced with fillers possessing extremely high thermal conductivity [54,55,56]. The percolation in the SR/BN and SR/EG composites occurred at filler volume fractions of 35% and 30%, respectively. The percolation in SR/EG occurred earlier than that in SR/BN, owing to the larger diameter of EG compared with BN.

**Figure 9:**
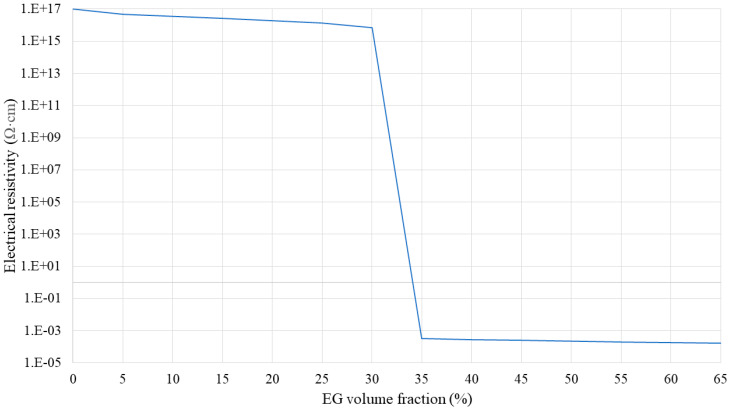
Electrical resistivity of the SR/EG layer as a function of the EG volume fraction.

**Figure 10:**
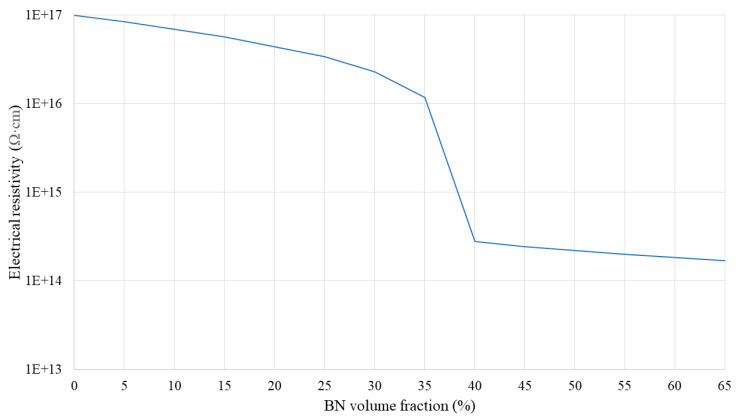
Electrical resistivity of the SR/BN layer as a function of the BN volume fraction.

**Figure 11:**
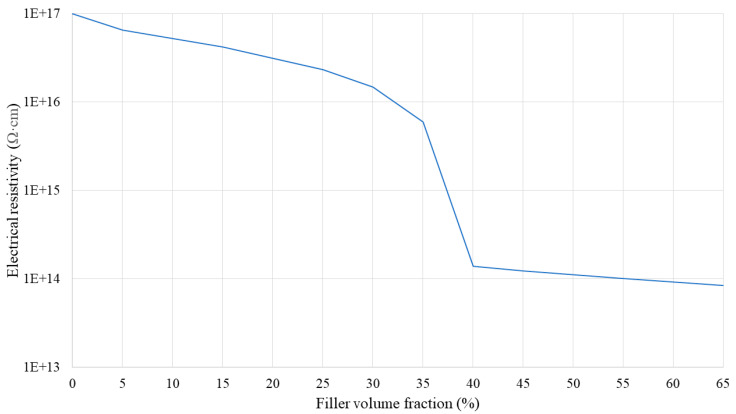
Electrical resistivity of the SR/BN-SR/EG composite as a function of the filler volume fraction.

Figure 9, Figure 10 and Figure 11 show the electrical resistivity values of SR/BN, SR/EG, and SR/BN-SR/EG as a function of the filler volume fraction in each layer, respectively. Similar to thermal conductivity, the electrical resistivity of the composite decreased sharply with changes in the filler volume fraction. However, the reduced values in the SR/BN and SR/BN-SR/EG frameworks did not reach the critical resistance for electrical insulation (10^9^ Ω·cm) [52]. The electrical resistivity of SR/EG decreased by nearly twenty orders of magnitude, dropping below 1 × 10^−3^ Ω cm at a filler volume fraction of 35%, indicating the material transition to an electrical conductor.

### 4.3. Effect of Filler Orientation

**Figure 12:**
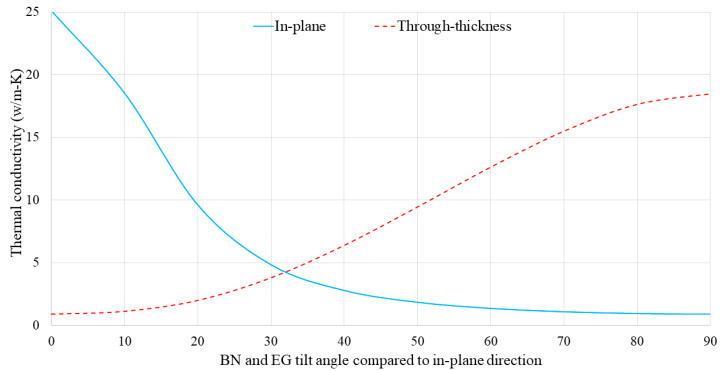
Through-thickness and in-plane thermal conductivities of the composite unit cell as a function of filler tilt angle.

As mentioned previously, the BN and EG fillers are highly aligned and oriented in the in-plane direction owing to the high shear forces generated by the two-roll mill during the manufacturing process. Thus, it was assumed that the BN and EG platelets were oriented and aligned in the in-plane direction. This assumption may have contributed to the discrepancy between the FE analyses and experimental results. Therefore, the in-plane and through-thickness thermal conductivities of the SR/BN-SR/EG composites were investigated at different BN and EG platelet orientations. In the FE modeling, the material orientation was controlled by adjusting the datum coordinate system of the composite unit cell at various angles. Figure 12 shows the results of the BN and EG tilt angles relative to the in-plane direction, with tilt angles of 0° and 90° indicating perfect horizontal and vertical alignment of the fillers, respectively. The in-plane thermal conductivity of the composite exponentially decreased as the filler tilt angle increased, reaching its lowest value at 90°. The in-plane thermal conductivity at 0° was more than 28 times that for 90°. In contrast, the highest through-thickness thermal conductivity was observed when the fillers were oriented at 90°. The through-thickness thermal conductivity at 90° was more than 21 times that for 0°. Notably, Chen et al. [45] observed this behavior in a polymer composite reinforced with BN. Additionally, Alghamdi [57] noted the same behavior in polymer composites with a differently shaped reinforcement, specifically, short carbon fibers.

### 4.4. Effects of Layer Configuration

**Figure 13:**
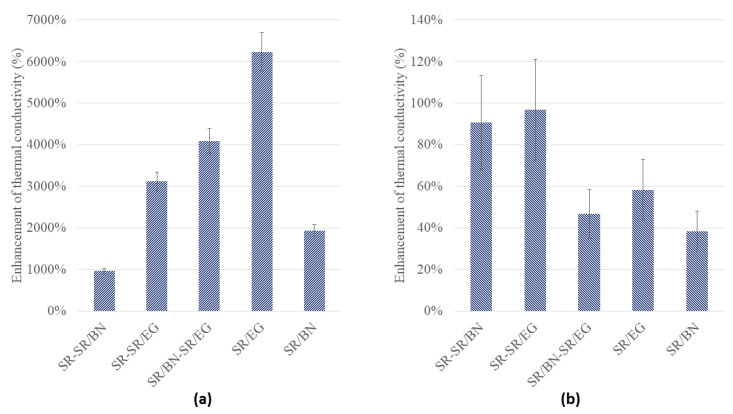
Enhancement in the (**a**) in-plane and (**b**) through-thickness thermal conductivities of SR composites with different configurations.

This thermal conductivity and electrical resistivity of the SR composites with different configurations, i.e., SR/BN-SR/EG, SR/BN, SR/EG, SR-SR/BN, and SR-SR/EG were compared. The volume fractions of BN and EG in the SR/BN and SR/EG frameworks were 0.395 and 0.35, respectively. Figure 13a,b shows the enhancement in the in-plane and through-thickness thermal conductivities of the SR composite with different configurations. The enhancement in the thermal conductivity of the SR composite was calculated as follows:

(5) $$Enhancement\;in\;thermal\;conductivity=\frac{K_{comp}-K_{SR}}{K_{SR}}\times 100,$$

where $K_{comp}$ and $K_{SR}$ denote the thermal conductivity of the composite and silicone rubber, respectively.

**Figure 14:**
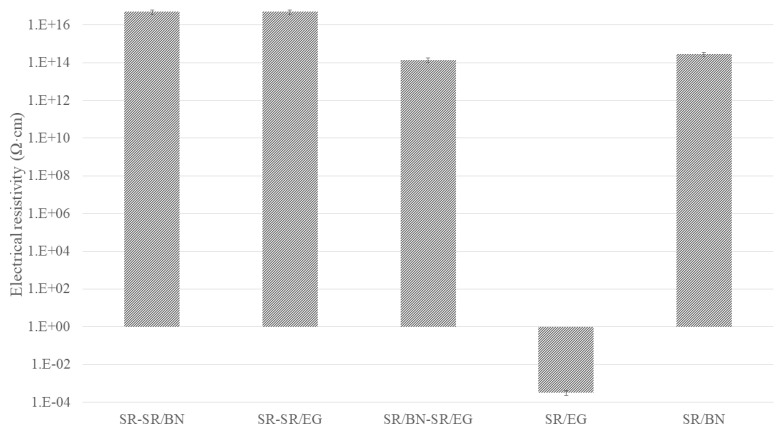
Electrical resistivity of SR composites with different configurations.

SR/EG exhibited the most substantial enhancement in in-plane thermal conductivity, exceeding 6000%, followed by SR/BN-SR/EG and SR-SR/EG, with enhancement percentages reaching 4000% and 3000%, respectively. Composites reinforced only with the BN filler presented the least enhancement (less than 2000% and 1000% for SR/BN and SR-SR/BN, respectively). Composites incorporating the SR layer achieved the highest enhancement in the in-plane thermal conductivity (97% and 91% for SR-SR/EG and SR-SR/BN, respectively). Despite the low thermal conductivity of SR, its isotropic properties facilitated through-thickness heat transfer. The through-thickness thermal conductivity of SR/EG, SR/BN, and SR/BN-SR/EG was enhanced by 58%, 38%, and 47%, respectively. Figure 14 shows the electrical resistivity of the abovementioned SR composites. All composites exhibited electrical resistivity exceeding the critical resistance for electrical insulation (10^9^ Ω·cm), except for the SR/EG composite, which demonstrated electrical resistivity lower than 1 × 10^−4^ Ω.

**Figure 15:**
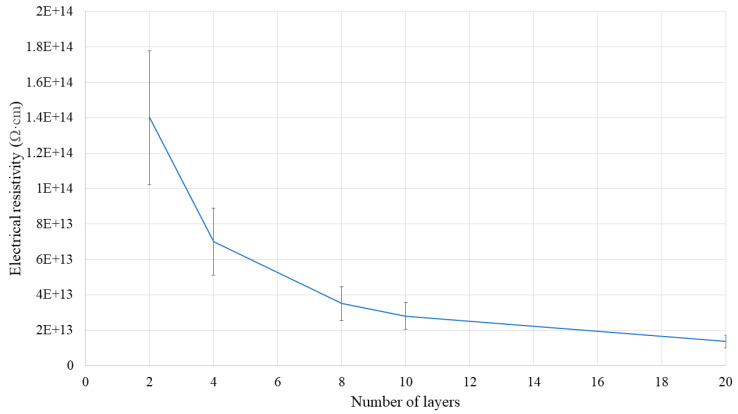
Electrical resistivity of SR/BN-SR/EG with different numbers of layers.

Figure 15 compares the electrical resistivity of SR/BN-SR/EG with different numbers of layers. Fewer layers corresponded to a higher electrical resistivity, and the increase in the electrical resistivity of the composite was exponentially proportional to the decrease in the number of layers. Xue et al. [36] derived similar conclusions for the same composite with four, six, and twelve layers.

## 5. Conclusions

A multiscale FE modeling technique was used to simulate the thermal conductivity and electrical resistivity of a multilayer SR composite reinforced with BN and EG. The effects of filler volume fractions and orientation on the thermal conductivity and electrical insulation properties were investigated. Additionally, the influence of the configuration and number of layers was explored. The following conclusions were derived:

- The thermal conductivity of the SR/BN-SR/EG composite was significantly influenced by the filler orientation.
- The in-plane thermal conductivities of SR/BN and SR/EG exhibited a percolation phenomenon characterized by a sudden rise in thermal conductivity at a critical volume fraction of the fillers, attributable to the formation of a filler network.
- Percolation in SR/BN and SR/EG occurred at filler volume fractions of 35% and 30%, respectively.
- A larger filler required lower volume fractions to achieve percolation in the in-plane thermal conductivity.
- The electrical resistivity of the SR/BN-SR/EG composite increased exponentially with a decrease in the number of layers.

## Figures and Tables

**Table 1:** Dimensions of the hexagonal filler unit cells for SR.BN and SR/EG layers.

Layer	Dimensions (µm)					
	*L*	*W*	*H*	*a*	*b*	*d*
SR/BN	8.66	12	0.475	8.66	0.3	10
SR/EG	17.32	24	0.178	17.32	0.1	20

**Table 2:** Material properties of composite constituents.

Phase	Thermal Conductivity		Electrical Resistivity	Source
	W⋅m^−1^⋅K^−1^		Ω·cm	
Boron nitride	62 (||)	1.5 (⊥)	≥10^13^	[46,47]
Expanded graphite	233 (||)	4.5 (⊥)	≤10^−5^	[48,49]
Silicone rubber	0.6		≥10^16^	[36,47]

**Table 3:** Thermal conductivities of different layers obtained using the hexagonal filler unit-cell model.

Layer	Thermal Conductivity (W⋅m^−1^⋅K^−1^)			
	Through-Thickness	In-Plane (x)	In-Plane (z)	In-Plane (Average)
SR/BN	0.83	22.56	1.86	12.21
SR/EG	0.95	74.11	1.93	38.02

**Table 4:** Comparison of thermal conductivities obtained using the composite unit-cell model and experiments.

Direction	Thermal Conductivity (W⋅m^−1^⋅K^−1^)		Difference withExperimental Results (%)
	Experimental	Finite Element Model	
Through-thickness	0.7 ± 0.01	0.88	25.71%
In-plane	23.4 ± 0.3	25.11	7.31%

**Table 5:** Electrical resistivity of different layers obtained using the hexagonal filler unit-cell model.

Layer	Electrical Resistivity (Ω·cm)	
	Discontinuous Filler	Continuous Filler
SR/BN	3.10 × 10^16^	2.80 × 10^14^
SR/EG	1.53 × 10^16^	3.16 × 10^−4^

**Table 6:** Electrical resistivity of the composite obtained using the composite unit-cell model.

	Electrical Resistivity (Ω·cm)	
	Discontinuous Filler	Continuous Filler
Composite	2.31 × 10^16^	1.40 × 10^14^

## Data Availability

Data are contained within the article.
